# Comparison of the touch-screen and traditional versions of the Corsi block-tapping test in patients with psychosis and healthy controls

**DOI:** 10.1186/s12888-020-02716-8

**Published:** 2020-06-23

**Authors:** Sara Siddi, Antonio Preti, Elvira Lara, Gildas Brébion, Regina Vila, Maria Iglesias, Jorge Cuevas-Esteban, Raquel López-Carrilero, Anna Butjosa, Josep Maria Haro

**Affiliations:** 1 Parc Sanitari Sant Joan de Déu, Fundació Sant Joan de Déu, Institut de Recerca Sant Joan de Déu, SantBoi de Llobregat, Universitat de Barcelona, Dr. Antoni Pujadas, 42, Sant Boi de Llobregat, 08830 Barcelona, Spain; 2 Centro de Investigación Biomédica en Red de Salud Mental (CIBERSAM), Instituto de Salud Carlos III, Madrid, Spain; 3 Psychiatry Branch, Centro Medico Genneruxi, Cagliari, Italy; 4 Center of Liaison Psychiatry and Psychosomatics, University Hospital, University of Cagliari, Cagliari, Italy; 5 Department of Psychiatry, Hospital Universitario de La Princesa, Instituto de Investigación Sanitaria Princesa (IIS-Princesa), Madrid, Spain; 6 Servei de Psiquiatria, Hospital Universitari Germans Trias i Pujol, Badalona, Catalonia Spain

**Keywords:** Working memory, Corsi task, Psychotic disorder

## Abstract

**Background:**

Working memory (WM) refers to the capacity system for temporary storage and processing of information, which is known to depend on the integrity of the prefrontal cortex. Impairment in working memory is a core cognitive deficit among individuals with psychotic disorders. The Corsi block-tapping test is a widely-used instrument to assess visuospatial working memory. The traditional version is composed of 9 square blocks positioned on a physical board. In recent years, the number of digital instruments has increased significantly; several advantages might derive from the use of a digital version of the Corsi test.

**Methods:**

This study aimed to compare the digital and traditional versions of the Corsi test in 45 patients with psychotic disorders and 45 healthy controls. Both groups completed a neuropsychological assessment involving attention and working memory divided into the two conditions.

**Results:**

Results were consistent between the traditional and digital versions of the Corsi test. The digital version, as well as the traditional version, can discriminate between patients with psychosis and healthy controls. Overall, patients performed worse with respect to the healthy comparison group. The traditional Corsi test was positively related to intelligence and verbal working memory, probably due to a more significant effort to execute the test.

**Conclusions:**

The digital Corsi might be used to enhance clinical practice diagnosis and treatment.The digital version can be administered in a natural environment in real-time. Further, it is easy to administer while ensuring a standard procedure.

## Background

Working memory (WM) deficit is a core cognitive dysfunction among people with Schizophrenia Spectrum Disorders [1]. Although WM deficits are well documented in psychosis, their magnitude and consistency vary depending on the tasks used [2]. For example, a meta-analysis by Lee and Park [2] reported WM deficits in both verbal and visuospatial tasks in individuals with schizophrenia. However, the latter deficits were more consistent and robust than those observed in verbal WM across studies. WM has also been noted as a predictor of occupational functioning [3] and has been related to improvements in psychological functioning following cognitive remediation [4] in individuals with psychotic disorders. Despite the extensive literature related to WM dysfunction in this population, there remains considerable debate regarding how best to measure this cognitive domain [5, 6].

In the Corsi block-tapping test [7], which consists of nine identical blocks on a board, participants are asked to repeat the sequence given by the evaluator in the same or reverse order. The evaluator taps the blocks in random sequences of increasing length. After the tapped sequence, the participant tries to mimic the tapping until he/she can no longer progress successfully. It is a simple yet powerful instrument to measure visuospatial working memory [8] and spatial attention [9]. It has frequently been used in individuals with various disorders: schizophrenia spectrum [10, 11], first-episode psychosis [12], and several neurological diseases [13–16].

In recent years, the number of neuropsychological tasks adapted to the digital context has increased significantly. Digital instruments for cognitive assessment offer several advantages, such as standardised automatic procedures and remote monitoring of health conditions in a natural, comfortable environment for the patients. This procedure allows information about health status to be obtained in real-time and in the real-world [17]. It is particularly important in people with psychosis, where the context can affect their emotional state during the execution of cognitive tasks [18].

In the traditional version of the Corsi block-tapping test, the evaluator can inadvertently change the presentation method by using a different finger to tap the blocks, by varying the speed of tapping, especially in longer sequences, or by covering some blocks during the presentation of the sequence. Moreover, in longer sequences (i.e., 8, 9 blocks), greater effort is required of the evaluators to remember the sequence during administration. Different computer-based versions of the original Corsi test have previously been tested in healthy children [19], in people with multiple sclerosis [13], and in schizophrenia spectrum disorders [11]. In the study by Girard et al. [11], participants were asked to repeat the sequence by clicking on the squares with a mouse, but this version involved different motor skills from those used in the traditional version of the test. In the computer version, the use of a mouse necessitates two interfaces: the vertical screen where the sequence is shown and the mouse which controls the cursor to tap the sequences. A preferable implementation of the Corsi test is on a tablet, as a touch screen is a single interface that the participant touches directly, just as with the physical board in the traditional version.

To the best of our knowledge, two previous studies [20, 21] have tested the validity of the digital Corsi test (d-Corsi) using the tablet in healthy individuals. Brunetti et al., [21] demonstrated the equivalence between the digital and traditional versions in a general population composed of younger and older adults. However, divergent and convergent validity, and known-group validity between people with psychosis and healthy controls have not yet been explored.

### Aims

The aims of the present study were: i) to test the validity of the d-Corsi test, with the expectation that no significant effect of the test version and no interaction between the test version and group effect would emerge ii) to explore the known-group validity between patients and healthy controls, with the expectation that both versions of the Corsi test would be able to distinguish the two groups, and iii) to investigate the convergent validity, with the supposition that the two versions would have similar correlations with verbal attention and working memory.

## Methods

### Sample

#### Patients

Forty-five patients with psychotic disorders (schizophrenia = 11, schizoaffective = 5; schizophreniform disorder = 4; unspecified psychotic disorder = 15; brief psychotic disorder = 1, delusional disorder = 1, affective disorders with psychotic symptoms = 8) were diagnosed by clinicians from the Parc Sanitari Sant Joan de Déu network of mental health services in Barcelona, Spain.

Inclusion criteria were: a) age between 18 and 65 years; b) a diagnosis of Schizophrenia Spectrum and Other Psychotic Disorders according to DSM-IV or DSM-5 criteria; c) fluency in Spanish, and d) ability to provide written consent. Exclusion criteria were: a) intellectual disability or cognitive impairments (IQ < 80 and Mini Mental State Examination (MMSE) < 25); b) a diagnosis of alcohol or substance abuse in the last 6 months; and c) neurological illness.

#### Healthy controls

The sample of healthy controls consisted of 45 adults from urban and suburban areas of Spain. Inclusion and exclusion criteria were the same as for the patient group; except for the diagnosis of mental disorders. A face-to-face interview was carried out to screen people for absence of current or previous psychiatric or neurological disorders and substance abuse or dependence, first-degree relatives of people with psychotic disorders, and intellectual disability (IQ < 80 and MMSE < 25). These screening questions were a condition of enrollment as healthy controls from the general population.

#### Ethics

The study protoco followed the guidelines of the 1995 Helsinki Declaration and subsequent revisions. The competent institutional review board of the Parc Sant Joan de Déu research committee and the Sant Joan de Déu ethics committee approved the study (PIC-64-16). Compensation was offered to all respondents for their participation (A *El Corte Inglés* gift card for 10€).

### Measures

#### Mini mental state examination test (MMSE)

This test was used as a screening tool to identify cognitive impairment. It consists of 30 items assessing orientation to time and place, memory registration, attention/calculation, memory recall, language, and visual spatial ability [22, 23]. Scores range from 0 to 30, with scores ≥25 interpreted as normal cognitive status.

#### Word accentuation test (WAT)

The WAT [24] is the Spanish version of the National Adult Reading Test [NART] [25]; which assesses premorbid IQ. Participants were asked to read aloud a list of 30 uncommon words without the stressed syllables marked. The total score is the sum of correctly read words.

#### Traditional Corsi block-tapping test (t-Corsi)

The traditional Corsi board structure consists of 9 blocks arranged irregularly on a 23 × 28-cm board [7]. The evaluator taps the blocks in sequences of increasing length (from 2 to 9 blocks) and two trials with different sequences (of equal length) are performed of each sequence. After each sequence, participants are required to tap the blocks in the same serial order. In case of the backward procedure, they are required to tap the blocks in the reverse order (i.e., from the last to the first block). Participants proceed to the following sequence (one item longer) if they reproduce the same sequence as the evaluator. The evaluator stops the test if a participant fails two trials of the same sequence or when the participant reaches the last sequence.

#### Digital-Corsi block-tapping test (d-Corsi)

**Fig. 1:**
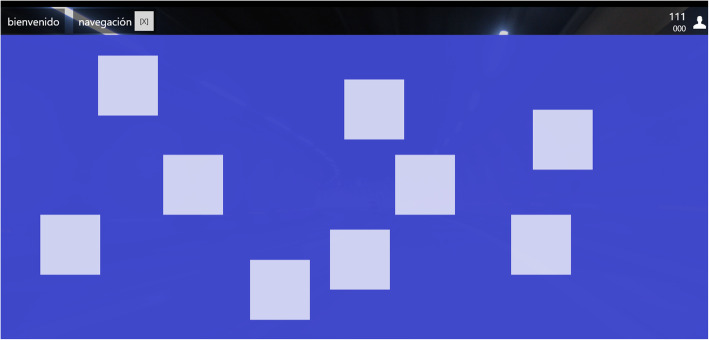
Digital-Corsi block-tapping test¨. A trial example of the Digital-Corsi block-tapping test

This software was developed by “SVEP srl”, Modena, Italy. It was installed on “Asus Transformer Book T100TA” tablets running *Windows* 8.1 NON RT. The Screen Width is 1920*0.08 (153 pixels) (please see Fig. 1). In the forward procedure, a sequence of blocks flashes on the tablet screen, each flash filling the square frame in red. Flashing time was set at 1000 ms. If the participants start tapping before the sequence is finished, the d-Corsi visually informs them to wait for the end of the sequence. The length of the sequences increases progressively as in the traditional version, starting with a sequence of 2 and then up to 9 squares. The test offers 10 levels of difficulty, tapping up to a sequence of 10 squares. However, in this study, participants could complete the task to the 9th level (9 squares).

Participants proceed to the following sequence (one item longer) if the reproduction is the same as that shown on the screen (or the reverse in the case of the backward procedure). As with the t-Corsi, two trials with different sequences (of equal length) are presented at each sequence. If the participant does not reproduce the sequence correctly, the system shows on-screen feedback using a visual warning indicative of an incorrect response, and ends the test automatically when all the sequences are completed or when two trials of a sequence are incorrect. Participant feedback might improve their performance in accuracy and speed. On the other hand, the feedback could also exert social pressure and increase lapses of memory. The whole test is managed by the software, which generates the sequences, the recording of the data (level and total score for forward and backward procedures), and cumulative reaction times. This software can work in two different modes: Quick mode (1 trial per level) and Manual Mode (2 trials per level). In this study, we used the manual mode to match the procedure used in the traditional version.

#### Digit-span test

The forward and backward digit spans of the Wechsler Memory Subscale (WMS) [26] were used to measure verbal attention and working memory, respectively. Digit sequences are presented, beginning with a length of two digits, and two trials are made at each increase of list length. The evaluator stops when the participant fails both trials of a sequence length or when the maximum list length is reached (9 for digit forward, 8 for digit backward).

#### Symptoms

In patients, the Positive and Negative Syndrome Scale (PANSS) Spanish version interview [27, 28] was used to assess positive and negative psychotic symptoms. The interview was conducted by clinicians involved in this study. Only attention deficit score was added to the analysis to explore the impact of general attention on the performance of the Corsi task in the patient group.

### Study procedure

Each participant completed forward and backward procedures of both Corsi block-tapping test versions (t-Corsi vs d-Corsi). The order of the versions was counterbalanced between the subjects. Between finishing the two versions of the Corsi test, each participant was also assessed on the above-mentioned instruments (WAT and digit span forward and backward).

### Statistics

#### Descriptive analysis

Frequencies and percentages were reported for categorical variables. Categorical analyses were made with the Chi-square test. Means with standard deviations were reported for continuous variables. Differences by groups in continuous variables were explored with Student’s t-test or ANCOVA, when appropriate. All tests were performed with SPSS version 20 [29] and R [30].

#### Comparison between individuals with psychotic disorders and healthy controls and correlation with t-Corsi and d-Corsi tests

A two-way repeated-measure ANCOVA was conducted on the Corsi scores to test if there are differences between the two versions. The within-subject factors were Corsi version (t-Corsi vs d-Corsi) and type of span (forward vs backward). Group (patients vs controls) and test administration order (A = 1st t-Corsi followed by d-Corsi; B = 1st d-Corsi followed by t-Corsi) were the between-subject factors. Reaction time was divided by the number of trials completed. Educational level was the control covariate for group differences in this measure.

General linear model (GLM), controlling for education, was computed to test the known-group validity and to explore which (t-Corsi and d-Corsi) distinguish better between patients and healthy controls. Partial eta-squared (η^2^) was used as a measure of effect size in the ANCOVA with 0.10, 0.25, and 0.40 considered as a small, medium, and large effect sizes, respectively.

#### Agreement between the t-Corsi and d-Corsi tests

The Bland and Altman [31] method was used to assess agreement between the two implementations of the Corsi block-tapping test (traditional and digital). The Bland-Altman plot visualizes the agreement between the scores of two different methods of assessment by plotting the difference between the two tests against the mean of two test scores for each participant. Confidence intervals for the mean difference are calculated to determine whether the mean difference deviates significantly from zero, which should not be the case. The plot draws the upper and lower limits of agreement, indicating the range within which 95% of the test scores in the two assessments can be expected to fall.

#### Network analysis between the t-Corsi and d-Corsi tests and other cognitive measures

The relationship between the Corsi block-tapping test in its two implementations (traditional and digital) was calculated with the Pearson correlation to test convergent and divergent validity. The relationship between attention deficits assessed with the PANSS interview and both versions of Corsi was also explored. Subsequently, the association between them and cognitive factors likely to affect performance (i.e., attention, working memory and premorbid IQ) was investigated with network analysis, which is a data-driven procedure that explores the links between variables by parceling out spurious correlations. In partial correlation networks, the association between two Corsi tests were computed after adjusting for the influence of all cognitive factors on the network [32]. The estimated links were further explored via a Gaussian Markov random field estimation using graphical LASSO (least absolute shrinkage and selection operator) and extended Bayesian information criterion to select the optimal regularization parameter. Calculations were made with the *bootnet* package running in R [33]. Graphical representation was made with the *qgraph* package running in R [34]. In this study, attention was measured with digit-span forward, working memory with digit-span backward, and premorbid intelligence with WAT (Spanish version of the NART). Composite reliability of the network was calculated by fitting a unidimensional confirmatory factor analysis model to each network and deriving reliability from the factor loadings. Differences across the three networks were tested with the van Borkulo Network Comparison Test [35]. The van Borkulo Network Comparison Test is an omnibus test that examines whether all edges are identical for each pair of networks. Post-hoc test, with Holm-Bonferroni method to correct for multiple testing, was applied to quantify how many of the estimated edges were different across each pair of networks [36].

## Results

### Baseline characteristics

Socio-demographics, neuropsychological functions and clinical characteristics of individuals with psychosis and healthy controls are summarized in Table 1. Patients and healthy controls were similar in terms of sociodemographic factors, except for educational level. Overall, patients performed worse on both versions of the Corsi test.

**Table 1:** Characteristics of patients and healthy controls

	Patients (***n*** = 45)	Healthy controls (***n*** = 45)	Statistics
Socio-demographic data			
Females, n(%)	18 (40)	18 (40)	NS
Age (years) mean (SD)	35.60 (11.53)	35.29 (11.08)	NS
Educational level ^b^ mean (SD)	3.33 (0.82)	5.11 (1.36)	t = 7.65 (72.019), *p* < 0.001
Hand laterality (right) n (%)	38 (84)	39 (87)	NS
Neuropsychological functions^a^ mean (SD)			
Premorbid IQ (WAT)	98 (8.38)	105 (6.35)	F = 1.82 (1,90), *p* = 0.170
Verbal attention (Digit-FW)	7.96 (2.09)	9.67 (2.19)	F = 2.76 (1,90), *p* = 0.069
Verbal working-memory (Digit-BW)	5.58 (2.17)	7.29 (2.37)	F = 3.02 (1,90), *p* = 0.054
MMSE	29.04 (0.97)	29.40 (0.80)	F = 0.44 (1,90), *p* = 0.640
Clinical characteristics			
Age of first hospitalization mean (SD)	28.07 (13.86)		
*Antipsychotics*			
Typical, n (%)	6 (13)		
Atypical,n (%)	56 (87)		
Mood stabilizer n (%)	12 (27)		
Antidepressantsn (%)	8 (18)		
Anxiolytics n (%)	40 (44)		
*PANSS*			
Positive symptoms mean (SD)(range)	17 (7.85) (7-35)		
Negative symptoms mean (SD) (range)	17.43 (6.44) (7-34)		
Attention deficit mean (SD) (range)	2.76 (1.11) (1-5)		

^a^ controlling for educational level. Note: ^b^ Educational level based on the following classification: 1 = no formal education; 2 = uncompleted primary education; 3 = completed primary education; 4 = uncompleted secondary school; 5 = completed secondary school; 6 = uncompleted university education; 7 = completed university studies. *NS* not significant, *SD* standard deviation, *FW* forward, *BW* backward, *MMSE* Mini Mental State Examination test, *WAT* Word Accentuation test, *PANSS* Positive and Negative Syndrome scale

### Equivalence between t-Corsi and d-Corsi

Table 2 displays means and standard deviations of forward and backward span for each version of Corsi. A high significant group effect was observed, reflecting lower memory scores in patients (F (1,85) = 10.19, *p* < 0.002, ^2^ = 0.11). No main effect of Corsi version emerged (F (1,85) = 0.03, *p* > 0.85), indicating that equivalent scores were achieved with the two versions. Neither Corsi version showed group interaction (F (1,85) = 0.58. *p* > 0 .44). Nor was effect of order of test administration observed (F (1,85) = 0.06, *p* > 0.80).

**Table 2:** Means and standard deviations of t-Corsiand d-Corsi tests

	n	t-Corsi FW		d-CorsiFW		t-CorsiBW		d-Corsi BW	
		M	SD	M	SD	M	SD	M	SD
Patients	45	7.56	2.18	7.60	1.85	6.73	2.01	6.64	2.56
Healthy controls	45	9.51	1.91	9.56	2.14	8.40	1.88	9.02	2.49
Procedure A	46	8.48	2.19	8.67	2.17	7.48	2.24	7.96	2.86
Procedure B	44	8.59	2.35	8.48	2.29	7.66	1.98	7.70	2.71
Patients (A)	23	7.78	2.27	7.74	1.81	6.91	2.35	6.91	3.11
Healthy controls (A)	23	9.17	1.92	9.61	2.12	8.04	2.03	9.0	2.19
Patients (B)	22	7.32	2.10	7.45	1.92	6.55	1.62	6.36	1.84
Healthy controls (B)	22	9.86	1.88	9.50	2.20	8.77	1.68	9.05	2.82
Patients’ timing^a^	45			8.19	6.82			7.09	2.95
Healthy controls’ timings^a^	45			6.74	3.09			7.31	5.25
All participants	90	8.53	2.26	8.58	2.22	7.57	2.11	7.83	2.78

Note: A = first t-Corsi test followed by d-Corsi test; B = first d-Corsi test followed by t-Corsi test; ^a^Reaction time divided by the number of trials completed

A further analysis (GLM) showed that the digital version was able to discriminate patients and healthy controls as well as the traditional version: d-Corsi (total) F (1,87) = 9.59, *p* < 0.003, η^2^ = 0.09; t-Corsi (total) F (1,87) = 6.73, *p* < 0.011, partial η^2^ = 0.07. No significant differences emerged between patients and healthy controls in the reaction time to perform the d-Corsi forward and backward.

### Agreement between t-Corsi and d-Corsi test

**Fig. 2:**
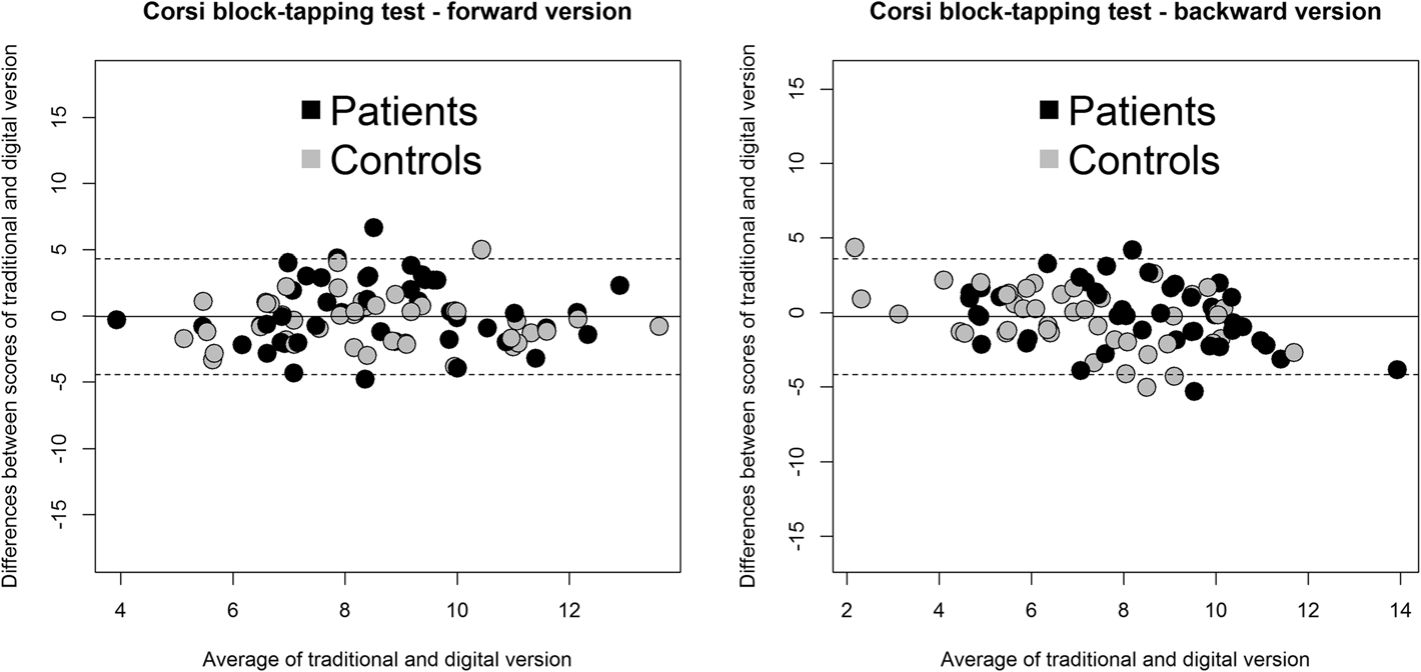
Agreement between the t-Corsi and d-Corsi block-tapping tests. Distribution of scores by version for the Corsi block tapping test in patients and healthy controls. Forward procedure on the right, and Backward procedure on the left. The upper and lower limits of agreement indicate the range within which 95% of the test scores in the two tests can be expected to fall

The two versions of the Corsi block-tapping test for both the forward and the backward measurements showed good agreement. Only three subjects were outside the upper and lower limits of agreement between the two versions of the test in the forward span, and only four in the backward span (Fig. 2).

### Correlation between the t-Corsi and d-Corsi test and other cognitive measures

The forward and backward spans of both versions of the Corsi test were positively related to each other with a high effect size (0.50). We observed a significant correlation between the two versions of Corsi measured with the Pearson correlation: t-Corsi forward and d-Corsi forward (*r* = 0.50, *p* < 0.001) and backward (*r* = 0.62, *p* < 0.001); and among t-Corsi backward and d-Corsi forward (*r =* 0.45, *p* < 0.001), d-Corsi backward (*r* = 0.70, *p* < 0.001). Significant associations between verbal attention and working memory tasks were also found (≥0.35). Attention deficit measured with the PANSS scale was positively related to t-Corsi forward (*r* = 0.37, *p* < 0.05) but not to d-Corsi tasks.

**Fig. 3:**
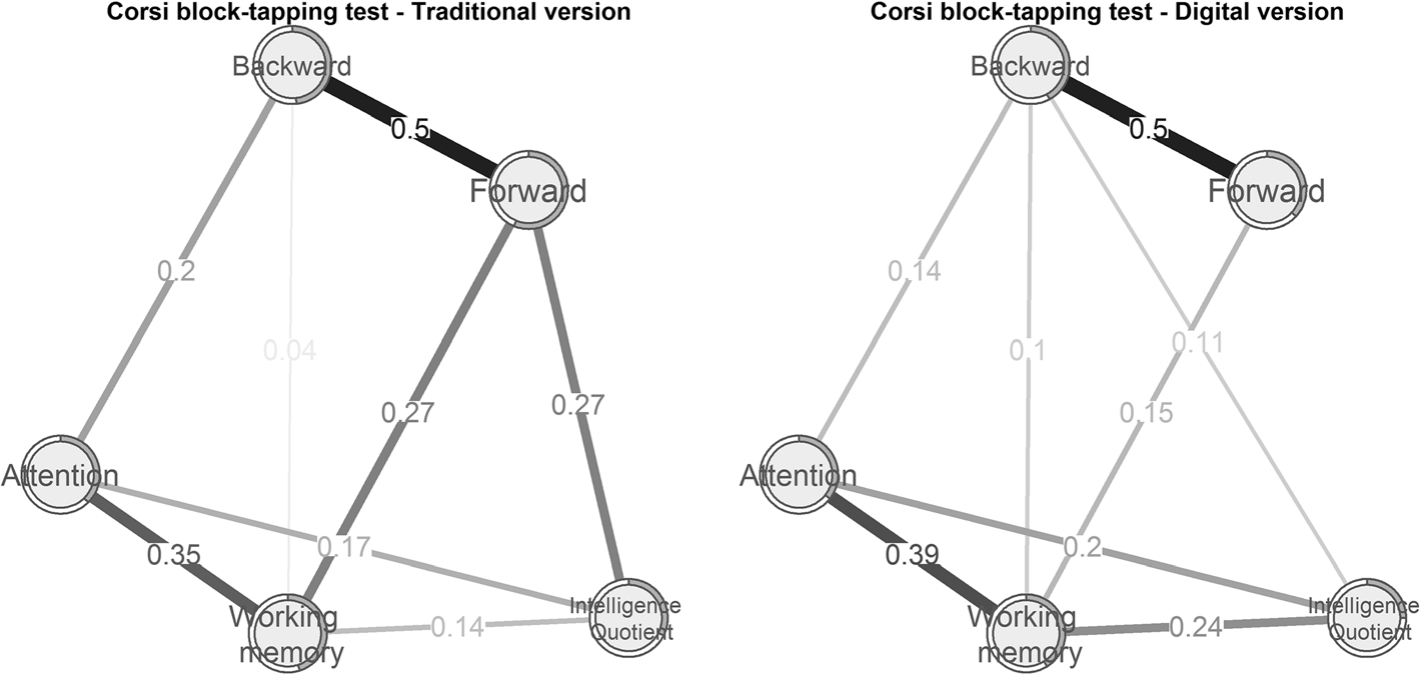
Network analysis between the t-Corsi and d-Corsiblock-tapping test and other cognitive functions. Network graph of the links among the Corsi block tapping test and three other cognitive functions: premorbid IQ, verbal attention, and working memory, in traditional (left) and digital (right) versions. Thickness of the lines is proportional to the estimated correlation coefficients, which are superimposed on the lines. Positive correlations are in “powder black”; negative correlations are in “grey”

Figure 3 summarizes the results of the network analysis. Composite reliability was acceptable in the networks of the traditional (0.82) and the digital (0.77) versions of the Corsi block-tapping test.

In the network, including the t-Corsi, IQ was positively related to the forward Corsi, which was also positively related to verbal working memory. In contrast, no association was found between IQ and the forward d-Corsi, and only a modest positive link between IQ, verbal attention, and the backward d-Corsi was observed. Despite these differences, the two networks did not differ from each other according to the van Borkulo Network Comparison Test: test statistic = 0.27, *p* = 0.19; and on global strength (a measure of association among variables): test statistic = 0.12, *p* = 0.47. None of the estimated edges was different across each pair of networks: Holm-Bonferroni corrected *p*-value per edge always remained above 0.20.

## Discussion

The findings confirmed that d-Corsi and t-Corsi are equivalents. The d-Corsi, as well as the traditional version, can distinguish between patients and controls. As expected, patients exhibited lower scores in forward and backward span of both types of Corsi test compared with healthy controls. No interaction effect was found per condition used. No feedback was provided during the traditional task. However, no differences emerged between the tasks. No significant differences emerged for the other tests. As we hypothesized, the span forward and backward of both types of Corsi test were positively associated with each other, as were verbal attention and working memory measured by the digit span. The t-Corsi was positively related to intelligence and verbal working memory. This link disappeared in the digital version. Overall, no relevant differences were found in the association of the Corsi test with cognitive factors that are likely to impact performance, in either its traditional or digital version.

### Agreement between t-Corsi and d-Corsi versions

The traditional and digital version have divergent accuracy patterns owing to the different procedures and characteristics of the tests (three-dimensional t-Corsi versus bi-dimensional d-Corsi). In the traditional version, the evaluator taps the block sequences, whereas the block sequence in the d-Corsi was indicated by the sequential lighting up of the various blocks.

The presentation duration of the block locations can be strictly applied in the digital version, whereas timing inconsistencies are likely to occur when an evaluator taps the sequences manually in the traditional version. In the d-Corsi, the evaluator is able to pay more attention to the behavior of the patient and the strategies that he or she applies to deal with the test, instead of being engaged with tapping the sequences with precise timing and examining the correctness of the patient’s responses [8].

The digital version is easy to install and is intuitive, and has additional advantages: accuracy in the presentation timing, absence of errors, and automatic score calculation, together with a standardized procedure and a user-friendly feel. The importance of standardization was clearly emphasized by Fischer [37], who showed that several test variables tend to influence performance levels.

### Known-group validity of d-Corsi test

The new d-Corsi test can distinguish patients from healthy controls as well as the traditional version. To the best of our knowledge, only one study has validated the digital version based on a computer modified version [11] showing that patients with psychotic disorders performed worse in the Corsi block Test than did healthy participants. Another study used a computerized version in the general population [38]. However, Woods et al. [38] used a computerized version in which the participants used the mouse to reproduce the sequence of blocks, which involves a distinct movement compared with the traditional version. Other studies have validated the Corsi test on a tablet device [20, 21] in healthy individuals. Different versions of this test have been made commercially available for the clinical and research fields, such as the Spatial Span subtest included in the WMS–III [26] and the Measurement and Treatment Research to Improve Cognition in Schizophrenia Consensus Cognitive Battery developed by the National Institute of Mental Health, useful in determining visual WM deficit in patients with schizophrenia. To the best of our knowledge, this Spatial Span subtest exists in a traditional but not in a digital version. Our test would be helpful in discriminating people at high risk of developing psychosis. The greater accuracy of the d-Corsi allows detection of mild cognitive impairments such as those found in subjects at high risk of psychosis.

### Convergent and divergent validity between t-Corsi and d-Corsi tests and other cognitive functions

Different strategies might be used by participants during the performance of traditional and digital tests. T-Corsi and d-Corsi were strongly associated and forward and backward conditions were strongly interrelated in both versions of the Corsi tests. On the other hand, it was observed that only t-Corsi forward was positively related to IQ and verbal working memory and attention (measured by PANSS). By contrast, we found a modest association among IQ, verbal attention, and the backward d-Corsi test. Participants might require greater verbal memory effort to reproduce the sequence in its original (forward) and reverse order (backward) in the traditional version compared with the digital. In d-Corsi, the participant only pays visual attention to the screen and has no need for the verbal instruction from the evaluator that is necessary in the t-Corsi version. Moreover, the evaluator may cover part of the blocks during the tapping of the sequence, thus reducing the visibility of all blocks. These conditions might interfere with the execution of the test. In this regard, Kessel et al. [39] maintained that verbal and visuospatial working memory are dissociated, possibly revealing the different cognitive processes that might underlie the two tests. Our findings are in agreement with previous studies in children [40] and older adults in the general population [41]. Brunetti et al. [42] suggested that the evaluator, during the sequence presentation of the t-Corsi, creates trajectories with hand-movement, and this might help the later performance of participants. In their study, they compared a digital version with trajectories (straight lines between each square during the presentation sequence) and a digital version without them. They found that this additional information enhanced the encoding of the stimuli.

#### Strengths and limitations

The d-Corsi is a valid, reliable assessment tool to evaluate visuospatial working memory in people with psychotic disorders. The d-Corsi showed different advantages in accuracy in timing presentation, standardized procedure, automatic score, and reaction time computation, and reduced risk of error. Moreover, the digitalization in a tablet enables the collection of large quantities of data in a quick, efficient manner, and thus allows the development of norms and cut-off scores that would be useful in a clinical context. The d-Corsi may require less effort for the evaluator and participants compared with the traditional version. This instrument could also be used outside the clinical context, for example, at home (i.e., experience sampling method). The information could then be used for neurorehabilitation.

Further studies should test the d-Corsi in different populations to confirm its consistency across diagnoses and cultures. It would be important to use the same version to be able to compare findings across studies. However, we should also acknowledge some limitations in our study: 1) the sample size was small; 2) the d-Corsi is more expensive than the traditional one due to the cost of the tablets; 3) the patients were undergoing treatment with antipsychotics, mood stabilizers, antidepressants, and anxiolytics; 4) a reduced battery of tests was used. Nonetheless, despite the cited limitation, the study was able to confirm the known-group validity, convergent and divergent validity of the d-Corsi.

## Conclusions

The d-Corsi might be used to enhance clinical practice diagnosis and treatment. Early detection of visuospatial working memory deficits linked to the risk of psychosis might be important to accelerate the development of prevenient interventions. This might be better appreciated in future studies in which psychosis risk is assessed in samples of help-seeking, high-risk people [43]*.*

## Data Availability

The datasets used and/or analyzed during the current study are available from the corresponding author on reasonable request.

## Abbreviations

ANCOVA: Analysis of covariance
d-Corsi: Digital Corsi block-tapping test
DSM: Diagnostic and Statistical Manual of Mental Disorders
GLM: General linear model
MMSE: Mini mental state examination test
NART: National Adult Reading Test
PANSS: Positive and Negative Syndrome Scale
t-Corsi: Traditional Corsi block-tapping test
WAT: Word accentuation test
WM: Working memory
WMS: Wechsler Memory Subscale
